# Analysis of the depression and anxiety status and related risk factors in patients with lumbar disc herniation

**DOI:** 10.12669/pjms.35.3.299

**Published:** 2019

**Authors:** Wenzhi Mu, Yong Shang, Chenchen Zhang, Shujie Tang

**Affiliations:** 1 *Wenzhi Mu Department of Functional Inspection, Yidu Central Hospital of Weifang, Qingzhou, Shandong province, 262500, China*; 2 *Yong Shang Department of Orthopaedics, Qingzhou Hospital of Chinese Medicine, Qingzhou, Shandong province, 262500, China*; 3 *Chenchen Zhang, School of Chinese Medicine, Jinan University, Guangzhou, 510632, China*; 4 *Shujie Tang, School of Chinese Medicine, Jinan University, Guangzhou, 510632, China*

**Keywords:** Lumbar disc herniation (LDH), Depression, Anxiety, Risk factors

## Abstract

**Objectives:**

To evaluate the depression and anxiety status and related risk factors in patients with lumbar disc herniation, and help spine surgeons better identify those patients who need psychological care.

**Methods:**

A cross-sectional study was performed on patients with lumbar disc herniation treated in our hospital between October 2015 and August 2018. Visual analog scale and Oswestry disability index were used to assess pain intensity and lumbar function, and Zung self-rating depression and anxiety scale were employed to evaluate the depression and anxiety status of the patients, and the demographic and clinical data including age, gender, marital status, occupation type, employment status, education level, surgery history, herniation type, disease duration, and insurance status were collected for analysis.

**Results:**

In the current study, 165 patients were enrolled based on the inclusion and exclusion criteria. In multivariate logistic regression analysis, gender (p=0.03), pain intensity (p=0.01), self-rating anxiety scale (SAS) (p=0.00), and disease duration (p=0.001) were identified as independent risk factors for depression status, and pain intensity (p=0.02), disease duration (p=0.002) and SDS (Zung self-rating depression scale) (p=0.003) were independent risk factors for anxiety status in patients with lumbar disc herniation. There was a significant correlation between Zung self-rating depression and anxiety scale in patients with lumbar disc herniation (p<0.05).

**Conclusion:**

Psychological intervention is critical for patients with lumbar disc herniation, especially for those female patients with severe pain and longer disease duration.

## INTRODUCTION

Low back pain (LBP), as one of the most common diseases, is being experienced by about 70% of people at some point in their life.^1^ It affects the quality of life in patients, and imposes a high economic burden on social health care system.^2^ Moreover, LBP is associated with psychological disturbance including depression and anxiety, which influence the outcomes of treatment adversely.^3^ Also, the improvement of the anxiety and depression status can help individuals control and manage pain in LBP.^4^ Lumbar disc herniation (LDH), which accounts for 50% of LBP patients, is the most common lumbar spinal disorder.^5^ Subsequently, psychological analysis should be paid high attention to in the treatment of LDH.

Currently, some studies have been conducted to assess the psychological status and determine the associated risk factors for abnormal psychology in LDH patients. Kim^6^ and D’Angelo^7^ reported depression or anxiety status was significantly associated with functional disability and pain intensity, and Chen found gender, pain intensity, and anxiety symptoms were risk factors for depression status, and disease duration and depression symptoms were risk factors for anxiety status.^8^ Sariyildiz and colleagues suggested that disease duration was not significantly correlated with the abnormal psychological status in LDH patients,^9^ but Guan advocated a different viewpoint.^10^ Obviously, these studies drew some conflicting conclusions, which may affect the psychological analysis and associated interventions for LDH patients. As a result, it is critical to perform a further study to evaluate the abnormal psychological status and related risk factors in patients with LDH.

Accordingly, we conducted this cross-sectional study on LDH patients treated in our hospital between October 2015 and August 2018’. The aim of this study was to evaluate the depression and anxiety status and related risk factors in LDH patients. We believe this study may help spine surgeons better understand the psychological status, and identify those patients who need psychological care.

## METHODS

This cross-sectional study was performed on LDH patients treated in our hospital between October 2015 and August 2018. The diagnosis of LDH was confirmed by clinical symptoms and computed tomography or magnetic resonance imaging examination. The inclusion criteria were: (1) patients diagnosed with LDH treated surgically or conservatively, and (2) patients who agreed to participate the study and signed informed consents. To facilitate the data analysis, only patients with one level herniation were included. Those patients with other pain syndromes, neurological disease, psychiatric disease as well as serious chronic disease such as cardiovascular disease, rheumatoid arthritis and epilepsy, which may interfere with the final outcomes, were excluded. The demographic and clinical data including age, gender, marital status, occupation type, employment status, education level, surgery history, herniation type, disease duration, and insurance status were collected for analysis. This study was approved by the ethics committee of our hospital.^11^

In this study, Visual analog scale (VAS) and Oswestry disability index (ODI) were used to assess the pain intensity and lumbar function in the enrolled patients. During the assessment of VAS, the patients recorded their pain on a sheet of 10-cm long paper divided by 10 lines. In VAS, pain is rated on a scale from 0 to10, in which 0 indicates no pain, but 10 indicates the severest pain which the patients can’t stand.^11^ ODI was used to evaluate the functional status of the lumbar spine, which consists of 10 items about daily living skills, each of which is scored from 0 to 5, the total scores are multiplied by two and expressed as a percentage, and higher scores indicate greater disability in patients.^11^ In addition, Zung self-rating depression scale (SDS) and self-rating anxiety scale (SAS) were used to evaluate the depression and anxiety status of the enrolled patients. Both scales are short self-administered surveys, each has 20 questions, each question is scored on a 4-point Likert scale, and 1 represents only a little of the time, and 4 represents most of the time^4^. The total scores of each scale range from 20 to 80, and the scores greater than or equal to 45 are defined as depression or anxiety status.

Statistical analysis was carried out using SPSS 21.0 (SPSS Inc., Chicago, IL, USA). The measurement data were compared by analysis of variance, and enumeration data by Chi square test. Multivariate logistic regression analysis was performed to determine the correlation between the demographic and clinical variables and depression or anxiety status. A p value less than 0.05 was regarded as statistical significance.

## RESULTS

In the current study, 165 patients were enrolled based on the inclusion and exclusion criteria. Among 165 patients, 116 were male and 49 were female, with an age ranging from 28 to 59 years old. The basic demographic, clinical and socioeconomic characteristics of the enrolled patients show in Table-I.

**Table-I:** The distribution of depression and anxiety status in the patients.

	Total number (n)	Anxiety (n)	Depression (n)
Number of patients	165	62	55
***Gender***			
Male	116	38	32
Female	49	24	23
***Age***			
≤50 years old	87	28	22
>50 years old	78	34	35
***Occupation type***			
Mental worker	106	42	38
Physical worker	59	20	17
***Employment status***			
Employed	149	59	50
Unemployed	16	3	5
***Marital status***			
Single	21	3	2
Married	108	41	39
Widowed	13	7	8
Devoiced	23	11	6
***Education level***			
Junior high school or below	15	2	3
Senior high school	68	26	24
University or above	82	34	28
***Insurance status***			
Yes	142	51	49
No	23	11	6
***Herniation type***			
Left rear herniation	65	20	18
Right rear herniation	63	23	21
Central herniation	37	19	16
***Surgery history***			
Yes	38	20	20
No	127	42	35
***Disease duration***			
≤ one year	93	27	21
> one year	72	35	34
***Vas scores***			
≤5	76	17	16
>5	89	45	36
***ODI scores***			
≤50	47	10	7
>50	118	52	45

In terms of depression status, 55 patients were included in depression group and 110 in non-depression group according to SDS scores, the rate of depression was 33.3%. There were significant differences in age (p=0.01), gender (p=0.029), surgery history (p=0.007), pain intensity(p=0.01), ODI scores (p=0.006) and disease duration(p=0.002), but no significant differences in occupation type (p=0.46), employment status(p=0.93), marital status(p=0.091), education level(p=0.609), herniation type(p=0.122), and insurance status(p=0.58) between the two groups (Table-I). In multivariate logistic regression analysis, gender (p=0.03), pain intensity (p=0.01), SAS (p=0.00), and disease duration (p=0.001) were determined as independent risk factors for depression status (Table-II).

**Table-II:** The independent risk factors for depression and anxiety in patients.

Variables	P value	OR	OR 95% CI	

			Lower	Upper
***Depression***				
Gender	0.03	6.281	1.745	29.501
VAS	0.01	4.672	1.985	10.462
SAS	0.00	11.213	4.701	45.681
Disease duration	0.001	7.955	2.391	19.872
***Anxiety***				
VAS	0.02	6.771	3.596	26.812
SDS	0.003	9.436	1.892	31.259
Disease duration	0.002	4.361	2.392	11.581

As to anxiety status, 62 patients were included in anxiety group and 103 in non-anxiety group based on SAS scores, the rate of anxiety was 37.6%. There were significant differences in marital status(p=0.012), surgery history (p=0.046), pain intensity(p=0.0003), ODI scores (p=0.01) and disease duration(p=0.015), but no significant differences in age (p=0.177), gender (p=0.07), occupation type (p=0.575), employment status(p=0.172), education level(p=0.144), herniation type(p=0.053), and insurance status(p=0.39) between the two groups (Table-I). In multivariate logistic regression analysis, pain intensity (p=0.02), disease duration (p=0.002) and SDS (p=0.003) were independent risk factors for anxiety status (Table-II).

**Fig.1:**
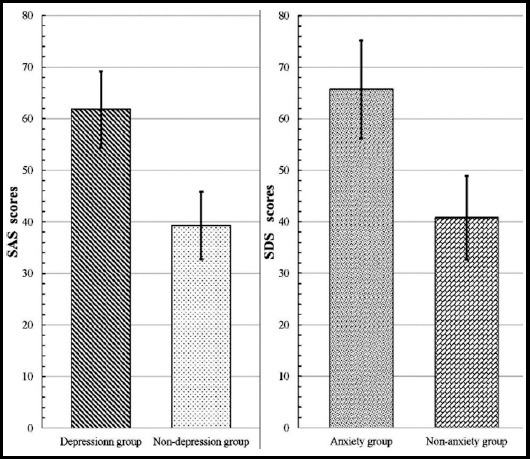
SAS and SDS scores in patients.

In addition, the mean SDS score in anxiety group and non-anxiety group was 65.7±9.5 and 40.8±8.1, respectively. There was significant difference (p<0.05) between the two groups (Fig.1). Similarly, the mean SAS score in depression group and non-depression group was 61.8±7.4 and 39.3±6.5, respectively. A significant difference (p<0.05) was detected between the two groups (Fig.1). Moreover, there was a significant correlation between SAS and SDS scores (p<0.05).

## DISCUSSION

In this study, we found that depression and anxiety in the enrolled patients was 33.3% and 37.6%, respectively, indicating the rate of LDH patients with abnormal psychological conditions was high, and these patients should be given more psychological care. Also, we confirmed female sex, higher SAS scores, higher pain intensity and longer disease duration were independent risk factors for depression, and higher pain intensity, longer disease duration and higher SDS scores were independent risk factors for anxiety in LDH patients, which may facilitate physicians better identify the patients with poor psychological conditions.

As to the risk factors for depression, in a study of 91 patients with LDH, Chen and colleagues concluded that female sex, SAS and VAS scores were independent risk factors,^8^ and in another study of 75 patients, Guan suggested that female sex and longer duration were independent risk factors.^10^ In above-mentioned two studies and our study, the only collectively recognized risk factor for depression was female sex. Gender is commonly regarded as an important factor related to depression, some scholars advocated that sex differences in pain perception exist in not only healthy subjects, but also LDH patients,^12^ female patients is more sensitive to pain, and subsequently, when suffered from severe pain, female patients may experience more depression. Severe pain and longer disease duration also produce more psychological stress, affecting the psychological status of the patients.^13^ However, it is noteworthy, in the three studies, some factors were not identified as risk factors for depression collectively, because the authors studied the issues from different angles, using different variables, and especially the sample size was different, leading to different conclusions.

In terms of the risk factors for anxiety, we found that pain intensity, disease duration and SDS scores were risk factors. Chen’s study also advocated that SDS scores and disease duration were independent risk factors, but pain intensity wasn’t.^8^ Compared with the present study, Chen’s study had a relatively small sample size, in our opinion, which may affect the statistical significance of pain. Moreover, some other studies also focused on the correlation between pain intensity and anxiety, in a study of 108 patients, D’Angelo found that the presence of trait anxiety before surgery was the main determinant of the presence of pain after surgery,^7^ which demonstrated the close correlation between anxiety and pain intensity from different perspectives.

Moreover, in this study we found there was a significant correlation between SAS and SDS scores, and depression and anxiety were risk factors for each other, they influenced each other and were reciprocal causation. At the same time, a study conducted by Edwards indicated that the symptoms of depression and anxiety were significant independent predictors of worse pain and function after controlling for relevant covariates.^14^ Subsequently, in this study we suggest again that psychological intervention is critical for patients with LDH, especially for those female patients with severe pain and longer disease duration, as they may experience more abnormal psychology.

### Limitations of this study

First, we confirmed some factors which were related to the depression or anxiety status in patients with LDH, but some other factors, such as bad lifestyle habits including tobacco use, physical inactivity and alcohol abuse, may also significantly affect the psychological conditions of patients, but these factors were not analyzed in the current study. Second, some socioeconomic factors, such as insurance and employment status, were not risk factors in the present study, but the influence of which on psychological status may be different in different areas or countries, as they are associated closely with socioeconomic conditions. Third, we studied the depression and anxiety status and related factors in this study, but some other abnormal psychology such as stubborn, hostility and somatization were not analyzed in this study, so the correlations between LDH and these abnormal psychological status as well as related risk factors are still unclear. Hence, more future studies need to be carried out to further clarify these issues.

### Authors’ Contribution

**SJT** conceived, designed and did statistical analysis & editing of manuscript.

**WZM, YS and CCZ** did data collection and manuscript writing.

**SJT and WZM** did review and final approval of manuscript.
